# House and Home

**Published:** 1893-11

**Authors:** Lillian White


### HOUSE AND HOME.

Conducted by Lillian White.

### THE HOUSE.

The Room and the Home —The divine law of harmony that permeates all nature is the one fundamental principle upon which it is safe to build, and wherever it is disregarded confusion is sure to result. No one thing should ever be purchased for itself alone. In relation to the room as a whole, its part in the projected scheme, its suitability to the use required, its adaptability as a factor, all are points to be studied and considered ; and then when such requirements are found, and intrinsic beauty is added as an extra charm rather than as an aggressive first thought, success will be attained and a well-nigh perfect selection will be made.

THE WORK BAG.—The favorite receptable for fancy work is not the dressy work-basket, but a dainty silken bag, with a basket bottom and long drawing strings, which can be thrown over the arm. The embroidery paraphernelia must all be of the finest—a gold thimble bearing one's initials, a fancy needle-book, a silver emery and a pair of silver embroidery scissors, a frame or a plush or a brocaded case for embroidery silks, itself a work of art and so eminently useful that it is worthy of description. The square of rich silk is made with a card-board centre. The lining may be the same silk or plain, interlined with cotton batting, sweet scented and feather stitched on inside edge. The corners fold over, handkerchief fashion, and are fastened with silken cords. There are inside straps, silk-covered and fastened at intervals to cardboard centres, to hold each colored skein in place and prevent them from getting tangled when not in use.

TABLE DECORATIONS.—Since the days when Eve spread her simple feast of tempting fruits before the angel Raphael we, her descendants, have striven to render the mighty name of dinner even more powerful by tasteful table decorations.

We have certainly made huge strides in the matter of taste during the past five decades, when the "festal board" was usually loaded with heavy vases and huge many colored "structures" of tightly compressed flowers. An attempt to gain a glimpse of one's opposite neighbor must have involved probable dislocation of the neck and certain loss of temper.

But we unhappy mortals are obliged to eat, and cannot, like the gods of Olympus, live on ambrosia and nectar, a charmingly economical, though slightly unsatisfying diet. Therefore we should try to make our food as appetizing as possible, and it cannot be denied that dainty surroundings materially assist good cookery.

FLOWERS AND VASES.—The decorative properties of a handsome vase are by no means to be despised. But all flowers do not show to advantage in vases. Others almost require them. Take roses. You cannot find a vase too gorgeous or too fine for them. They can be put even in the ponderous jars that stand shoulder-high on the floor.

But if you have only a few roses it is well to discriminate. You often have a dozen or so, and you are in the habit of putting them all together in the prettiest vase. But does not the doing this often cause you pain?

One rose is so sadly crushed by the operation. Another has its prettiest side turned away. A third has to part with some of its leaves, may be. And to-morrow all will wilt togetner. It is a disappointing pity.

But try this way. Take the very best rose in the lot and put it in a vase by itself. If you have not a long, narrow-necked rose vase get one when you can. A dollar will buy two small Japanese ones of that kind. A rose is far too fine to hide its beauty and fragrance under a bushel of others.

Very long-stemmed "Jack" roses and American Beauties may be bunched. Their stems are long enough to allow each rose to rear its head proudly aloft to be admired by itself. Round cut-glass rose jars come in for holding a bunch of roses. They can be bought for from thirty-five cents for the small, common glass ones to as many dollars for the big ones in cut-glass.

### THE DRESS.

A Day Dress.—A day dress for country wear is of electric blue cloth, bordered on the skirt with a black and gold check velvet band, opened on each side with revers of blue over the checked velvet. The waist is held by a black velvet band, and a pelerine of black lace fastened by a broad band of jet adorns the neck of the bodice. In all toilets whether for house or street wear, the sable boa is very much used, and is a great protection from cold in unheated apartments.

One characteristic of the present season is the expensive nature of the materials used in dresses. Velvets, silks and satins are in general use, and some of the smartest dresses are made of moire antique. Mirror velvets are still worn, and are often spotted, while spotted silks and satins are very much to the front. The richest and thickest point lace is in demand for trimmings, and with this go such expensive furs as ermine, sable, beaver and chinchilla. Many very beautiful dresses are made in satin of the rich ordinary kind and the reversible when the two shades serve mutually to enhance each other's effect.

Stylish Jacket.—Every jacket has immense sleeves and the flaring skirt that gives them a new and entirely different look from those on view last season. The handsomest model in this form was a Parisian fancy in black broadcloth, with six deep tucks in the skirts. The body was tight-fitting and had a beautiful collar of broad tail, a fur which is almost if not quite as expensive as sealskin, it being the covering of the unborn Persian lamb. The sleeves in this jacket were of eminence purple velvet, full at the shoulders and narrowing down to very slender proportions in the forearm.

STYLISH HAT.—Hats, consisting of a perfectly flat, plate-like piece, mounted on a round band and trimméd with a single upright bow of ribbon that is tied right through the hat itself and stiffly wired, are among the new things. They are stylish, and the more impossibly tall and stiff the bow is and the more the flatness of the hat makes its security a matter of amazement, the more stylish it seems to be.

### THE KITCHEN.

CELERY SAUCE.—Cut celery stalks into inch lengths, stew in butter until they are tender. Add a tablespoon of flour, allow it to brown a little, and then pour in half a pint of good broth or beef gravy. Season with a little white pepper and a dash of celery salt.

EGG CUTLETS.—For egg cutlets take four eggs boiled hard ; into a saucepan put one tablespoonful of butter and two tablespoonfuls of flour. Stir over the fire without browning, adding half a pint of milk, one level teaspoonful of salt, half a teaspoonful of pepper and the chopped whites of the eggs, with one tablespoonful of chopped parsley. Make into small cutlets, using for each the unbroken yolk covered with the mixture. Dip in egg and bread crumbs and fry.

ROASTED CHEESE.—Grate three ounces of rich cheese, mix with the yolks of two eggs, four ounces of grated bread, three ounces of butter ; beat all together well in a mortar, with a dessertspoonful of mustard and a little pepper and salt. Toast some bread, cut it into proper pieces, lay the paste as above thick upon them, put into an oven, covered with a dish, till hot through ; remove the dish, and let the cheese brown slightly. Serve as hot as possible.

SCALLOPED POTATOES.—Butter the bottom and sides of a dish. Put in a layer of cold boiled potatoes, sliced, season with pepper, salt and small pieces of butter and dust with flour. Put in another layer of potatoes in the same way, and when the dish is filled cover the top with a layer of cracker crumbs half an inch thick. Pour a cup of cream over the whole, and bake in a moderate oven for half an hour. This may be varied by the use of a seasoning of finely chopped celery or parsley.

BANANA SHORT CAKE.—Cream one-half cup butter, one cup of sugar, stir in one beaten egg, half a cup of milk, two cups of flour and two teaspoonfuls of baking powder. Bake in round or oblong tins. Over one cake spread a pint of whipped cream. Sweeten to taste, into which has been stirred one large banana sliced thin. Lay the other over it and serve very hot.

FRIED CHICKEN.—Clean and cut the chicken the same as for a fricassee. Dredge each piece thickly with salt, pepper and flour. Put three tablespoonfuls of oil or lard in a frying pan ; and when very hot, put in the chicken, and fry slowly until it is done. If young (as it should be), it will fry in three-quarters of an hour. Watch it carefully that it may not burn. When done, arrange the pieces on a hot dish. Pour all the fat, but about one tablespoonful, from the frying-pan ; then add a tablespoonful of flour, mix and add a half-pint of milk or cream, stir, season with salt and pepper, and pour over the chicken.

HOMINY MUFFINS.—For twelve muffins use one cupful of warm boiled hominy, one cupful of milk, one cupful and a half of flour, one generous teaspoonful of baking powder, half a teaspoonful of salt, one tablespoonful of butter, one teaspoonful of sugar and two eggs. Mix all the dry ingedients and rub them through a sieve. Beat the butter into the hominy and gradually beat in the milk. Beat the eggs till very light. Add the hominy and milk to the dry ingredients and beat well ; then add the well beaten eggs. Pour the batter into buttered muffin pans and bake in a rather hot oven for half an hour.

---

### LEAVE TAKING.

Not all have learned the fine art of leave taking in an appropriate manner. When you are about to depart, do so at once, gracefully and politely, and with no dallying. Don't say, "It's about time I was going," and then settle back and talk on aimlessly, for another ten minutes. Some people have just such a tiresome habit. They will even rise and stand about the room in various attitudes, keeping their host also standing, and then by an effort succeed in getting as far as the hall, when a new thought strikes them. They brighten up visibly and stand for some minutes longer, saying nothing of importance, but keeping every one in a restless, nervous state. After the door is opened the prolong leave taking begins, and everybody in general and particular is invited to call. Very likely a last thought strikes the departing visitor, which his friend must risk a cold to hear to the end. What a relief when the door is finally closed! There is no need of being offensively abrupt, but when you are ready to go—go.

It may be true that women spend nine-tenths of the money in the world, but the greater part of it goes to buy things for the children and the men.

It is a distinct advance for a man to acquire the ability to say, on occasion, "I can't afford it;" but he ought to say it just as often to himself as he says it to his wife.

